# Transcriptome-based repurposing of apigenin as a potential anti-fibrotic agent targeting hepatic stellate cells

**DOI:** 10.1038/srep42563

**Published:** 2017-03-03

**Authors:** Daniel F. Hicks, Nicolas Goossens, Ana Blas-García, Takuma Tsuchida, Benjamin Wooden, Michael C. Wallace, Natalia Nieto, Abigale Lade, Benjamin Redhead, Arthur I Cederbaum, Joel T. Dudley, Bryan C. Fuchs, Youngmin A. Lee, Yujin Hoshida, Scott L. Friedman

**Affiliations:** 1 Division of Liver Diseases, Department of Medicine, Liver Cancer Program, Tisch Cancer Institute, Graduate School of Biomedical Sciences, Icahn School of Medicine at Mount Sinai, New York, USA; 2 Division of Gastroenterology and Hepatology, Geneva University Hospital, Geneva, Switzerland; 3 Department of Pharmacology, University of Valencia-FISABIO, Valencia, Spain; 4 Research Division, Mitsubishi Tanabe Pharma Corporation, Saitama, Japan; 5 University of Western Australia, West Leederville, WA, Australia; 6 Department of Pathology, University of Illinois at Chicago, Chicago, USA; 7 Department of Genetics and Genomics Sciences, Icahn School of Medicine at Mount Sinai, New York, USA; 8 Department of Pharmacology and Systems Therapeutics, Icahn School of Medicine at Mount Sinai, New York, USA; 9 Division of Surgical Oncology, Massachusetts General Hospital Cancer Center, Harvard Medical School, Boston, USA

## Abstract

We have used a computational approach to identify anti-fibrotic therapies by querying a transcriptome. A transcriptome signature of activated hepatic stellate cells (HSCs), the primary collagen-secreting cell in liver, and queried against a transcriptomic database that quantifies changes in gene expression in response to 1,309 FDA-approved drugs and bioactives (CMap). The flavonoid apigenin was among 9 top-ranked compounds predicted to have anti-fibrotic activity; indeed, apigenin dose-dependently reduced collagen I in the human HSC line, TWNT-4. To identify proteins mediating apigenin’s effect, we next overlapped a 122-gene signature unique to HSCs with a list of 160 genes encoding proteins that are known to interact with apigenin, which identified *C1QTNF2*, encoding for Complement C1q tumor necrosis factor-related protein 2, a secreted adipocytokine with metabolic effects in liver. To validate its disease relevance, *C1QTNF2* expression is reduced during hepatic stellate cell activation in culture and in a mouse model of alcoholic liver injury *in vivo*, and its expression correlates with better clinical outcomes in patients with hepatitis C cirrhosis (n = 216), suggesting it may have a protective role in cirrhosis progression.These findings reinforce the value of computational approaches to drug discovery for hepatic fibrosis, and identify *C1QTNF2* as a potential mediator of apigenin’s anti-fibrotic activity.

The hepatic stellate cell (HSC) represents the major focus for developing anti-fibrotic therapies, whereas other cell types, e.g, portal fibroblasts, also contribute to fibrogenesis to a minor extent depending on the site, duration and nature of liver injury^1^,^2^. HSC-specific genes and proteins likely serve as clues to candidate therapeutic targets and will accelerate preclinical testing and clinical development of anti-fibrotic therapies.

Bioinformatic interrogation of public databases is a comprehensive and efficient strategy to identify disease molecular signatures, drug targets, and even candidate drugs. There are a growing number of transcriptome datasets available for the identification of genes and pathways unique to a variety of biological and clinical contexts across multiple assay formats and tissue types, including liver^3^. Liver disease-specific molecular signatures such as regulators of collagen deposition and hepatocellular carcinoma subtype/prognosis classifiers have been identified to date^4^,^5^,^6^,^7^. Our previous unbiased interrogation of the liver cell transcriptome compendium has identified a 122-gene HSC-specific molecular signature uniquely expressed in quiescent and/or activated HSCs compared to other cell types in the liver, which was associated with poorer clinical outcome in patients with hepatitis C virus (HCV)-related cirrhosis and hepatocellular carcinoma^8^.

In parallel, molecular signature-based *in silico* drug screening and repurposing has been successfully utilized for quick hypothesis-free identification of novel therapeutics for a variety of cancers and inflammatory diseases, among many others diseases^3^. In the current study, we used an experimentally-defined HSC activation gene signature in an unbiased manner as a basis for applying a computational drug discovery approach to identify candidate anti-fibrotic drugs that antagonize the HSC gene signature.

## Material and Methods

### Computational compound screen for candidate anti-fibrotic agents

An HSC activation signature was defined in transcriptome profiles of freshly isolated HSCs from cirrhotic rat liver treated with repeated low-dose diethylnitrosamine (low-dose DEN rat)^9^ (NCBI Gene Expression Omnibus [GEO] accession number, GSE63726). Differentially expressed genes between the cells isolated from cirrhotic and healthy control livers were defined after making to human orthologous genes (NCBI HomoloGene database, release 68) by random permutation t-test based on significance threshold of false discovery rate (FDR < 0.05) (Supplementary Table 1). The gene signature was used to query a database of transcriptome profiles of 1,309 unique FDA-approved drugs and bioactive compounds, the Connectivity Map (CMap) database (https://portals.broadinstitute.org/cmap/)^10^. Compounds with significant negative association (enrichment p ≤ 0.05) were selected as candidates for subsequent experimental evaluation.

### *In vitro* assessment of candidate anti-fibrotic agents

TWNT-4 (human HSC line) cells^11^ were seeded onto a 96-well plate at 5,000 cells per well in 100 μl of assay medium (DMEM, 10% FBS, 1% penicillin/streptomycin), and cultured at 37 °C and 5% CO_2_. After 24 hours, the cells were treated with apigenin (Sigma-Aldrich) (≥97% purity) at final concentrations of 2.5 μM, 10 μM, 20 μM, 40 μM, 60 μM, 80 μM, 100 μM, and 200 μM dissolved in 0.5% DMSO or DMSO control in triplicate for 24 hours. Cell viability was measured by MTS assay using CellTiter 96^®^ Aqueous One Solution Reagent (Promega) following manufacturer’s instruction. Percentage of mean absorbance of each drug-treated condition over the control was calculated.

### Quantitative reverse transcriptase polymerase chain reaction (qRT-PCR)

RNA was extracted from adherent cells using RNeasy Mini kit (Qiagen). Equimolar concentrations of RNA were converted to cDNA (Clontech), and quantitative real-time PCR was performed using SYBR green reagent (Roche) on the Lightcycler 480 system (Roche). Gene expression level in each sample was internally normalized to *GAPDH* expression. The following PCR primers were used (5′ to 3′): GGCTTCCCTGGTCTTCCTGG (forward) and CCAGGGGGTCCAGCCAAT (reverse) for human *COL1A1*; GAGGCTCCTCCCAGTCATCA (forward) and GGGATCATGGTGGTTACCCAGA (reverse) for human *C1QTNF2*; CCAGAAGCCATCAGCAGCAAG (forward) and AGGCCCTGAGAGATCTGTGG (reverse) for human *PDGFRB*: AGGCACCCCTGAACCCCAA (forward) and CAGCACCGCCTGGATAGCC (reverse) for human *ASMA*; CAAGGGCTACCATGCCAACT (forward) and AGGGCCAGGACCTTGCTG (reverse) for human *TGFB1*; CGAGTGCCAAATGAAGAGGACC (forward) and AAACCTGAGCCAGAACCTGACG (reverse) for human *TGFRB1*; CAATGACCCCTTCATTGACC (forward) and GATCTCGCTCCTGGAAGATG (reverse) for human *GAPDH*.

### Western blotting

TWNT-4 cells were lysed in RIPA buffer (150 mM NaCl, 50 mM Tris-HCl, 1% IGEPAL, 0.5% Sodium deoxycholate, 1% SDS) with proteinase inhibitors (Roche) and pelleted. Inguinal adipose tissue was dissected from two C57BL/6 mice (Charles River). Whole liver tissue was isolated from 5 control mice fed a normal diet for 6 weeks and 5 mice fed a Lieber DeCarli ethanol-containing diet for 6 weeks. In both cases, the tissue was lysed mechanically using steel beads in a TissueLyser LT (Qiagen) and with RIPA lysis buffer with the proteinase inhibitor. The lysate was sonicated and pelleted and the aqueous supernatant was isolated. Twenty μg of protein from each sample was suspended in NuPAGE LDS sample buffer and heated for 10 min at 70 °C. Samples were electrophoresed on 10% BisTris NuPAGE gels (Invitrogen) and then transferred to nitrocellulose membranes (Invitrogen). Membrane blotting was performed using the following primary antibodies, rabbit polyclonal anti-COL1A1 antibody (Rockland, Limerick, PA, catalog #600-401-103) (1:5000), rabbit polyclonal anti-C1QTNF2 antibody (ProSci, catalog #3561) (1:1000), mouse monoclonal anti-GAPDH antibody (Millipore, catalog #CB1001) (1:2500), mouse monoclonal anti-β-tubulin (Sigma-Aldrich, catalog #T4026) (1:2500), mouse anti-calnexin (Abcam, catalog #75801) (1:2500) and appropriate HRP-conjugated secondary antibody. Bands were visualized with chemiluminescent HRP antibody detection reagent (HyGlo e2400, Denville), captured with Amersham Imager 600 (GE Healthcare Life Sciences), and quantified using ImageJ software (https://imagej.nih.gov/ij/).

### Immunofluorescence staining

A total of 50,000 TWNT-4 cells were plated onto glass coverslips and cultured until 90% confluent, and then fixed with 100% acetone for 10 minutes at −20 °C. The cells were subsequently permeabilized in Tween-20 detergent in PBS for 20 minutes and then incubated in the rabbit polyclonal anti-C1QTNF2 antibody (1:1000) with negative and positive controls, chicken polyclonal anti-GFAP (Abcam, catalog #4674) (1:200), and anti-Desmin (AbCam, catalog #15200) (1:200). Appropriate green fluorescent tagged secondary antibodies (Life Technologies) were used and DAPI was used for nuclear staining. Cells were imaged under Eclipse TS100 fluorescent microscope (Nikon).

### Culture activated mouse HSCs

DNA microarray-based transcriptome profiles of mouse primary HSCs before (day 0) and after *in vitro* culture activation (day 7) were obtained from GEO database (NCBI Gene Expression Omnibus [GEO] accession number, GSE34949)^12^,^13^.

### Clinical HCV cirrhosis cohort

DNA microarray-based transcriptome profiles of 216 patients with HCV-related compensated cirrhosis we previously reported were used to evaluate prognostic association of *C1QTNF2* expression level (GSE15654)^6^. *C1QTNF2*-high.group was defined as samples with *C1QTNF2* expression higher than one standard deviation above mean. Prognostic association was assessed by Kaplan-Meier curve and log-rank test.

### Statistical analysis

Continuous values are presented by mean and standard error of mean (SEM). Differences were assessed by either t-test or one-way ANOVA followed by Dunnett’s multiple comparisons test. Two-tailed p-value less than 0.05 was regarded as statistically significant. All statistical analyses were performed using Graph Pad Prism version 7.0a (GraphPad Software).

## Results

### Computational screen to identify candidate anti-fibrotic agents

A 673-gene *in vivo* HSC activation signature was defined in the isolated HSC fraction from the low-dose DEN rat (Supplementary Table 1). The gene signature was used to query the compound perturbation transcriptome database (CMap) for candidate anti-fibrotic agents that potentially antagonize the HSC activation signature. Eighteen compounds with significant negative association (p ≤ 0.05) were identified (Table 1). Of note, the majority of the compounds (n = 14, 78%) are not recognized for their possible anti-fibrotic effect, highlighting the potential advantage of this unbiased in silico screen to efficiently identify candidate drugs. Among them, 9 top hit compounds commercially available and without clinically known severe toxicity were chosen for subsequent experimental evaluation.

### *In vitro* validation of anti-fibrotic effect of apigenin in a HSC cell line

**Figure 1:**
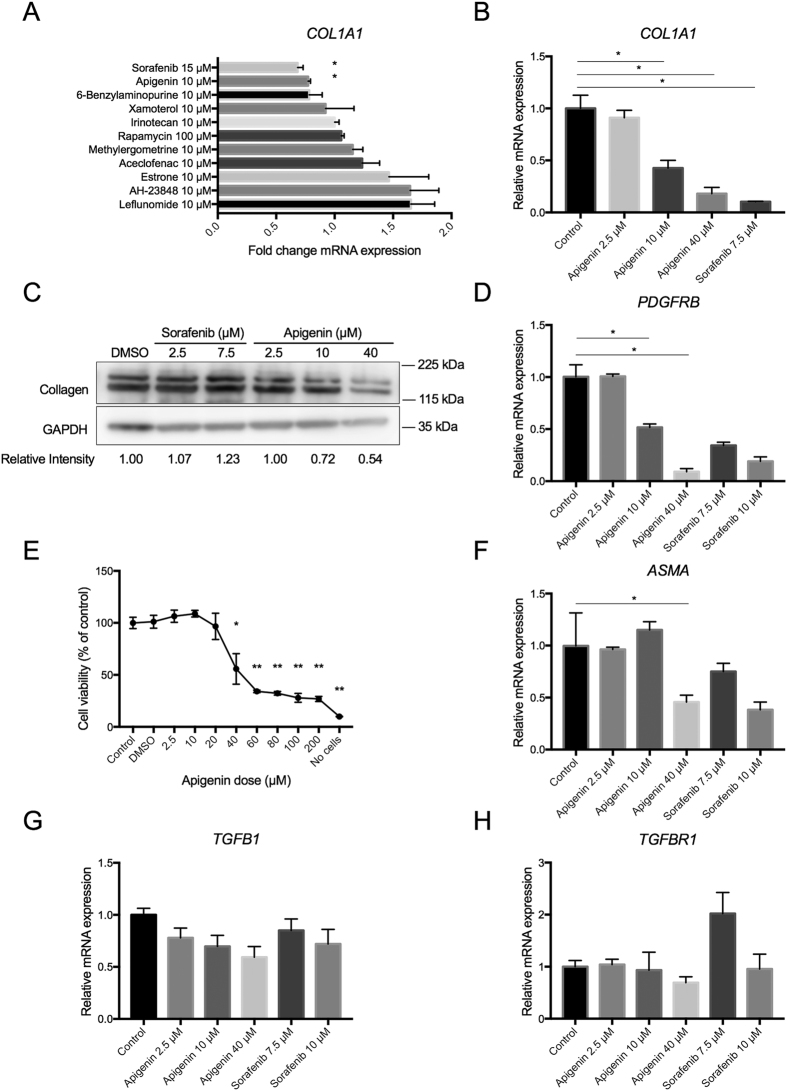
Anti-fibrotic effect of apigenin in human HSC line (TWNT-4 cells). (**A**) Modulation of *COL1A1* expression by nine computationally selected candidate compounds together with sorafenib and rapamycin (positive controls) compared to DMSO-treated controls (qRT-PCR, n = 3). *p < 0.05, t-test. (**B**) Dose-dependent suppression of *COL1A1* expression by apigenin (qRT-PCR, n = 3). *p < 0.001, Dunnett’s test. (**C**) Suppression of collagen 1 protein by sorafenib and apigenin (Western blotting, n = 1). Relative intensity to GAPDH was calculated. (**D**) Modulation of *PDGFRB* expression by apigenin and sorafenib (qRT-PCR, n = 3). *p < 0.001, Dunnett’s test. (**E**) Cell viability in association of apigenin dose (MTS assay, n = 3). *p < 0.01, **p < 0.001, Dunnett’s test. (**F**) Modulation of *ASMA* expression by apigenin and sorafenib (qRT-PCR, n = 3). (**G**) Modulation of *TGFB1* expression by apigenin and sorafenib (qRT-PCR, n = 3). (**H**) Modulation of *TGFRB1* expression by apigenin and sorafenib (qRT-PCR, n = 3). Bar graphs show mean and standard error of mean (SEM) (error bars) of replicated experiments.

The 9 computationally prioritized candidate compounds were tested for their anti-fibrotic effect in a human HSC cell line, TWNT-4, together with a multi-kinase inhibitor, sorafenib, and an mTOR inhibitor, rapamycin, as positive controls^14^,^15^. Apigenin, a flavonoid with a known anti-fibrotic activity in a mouse model of chronic pancreatitis^16^,^17^, was the only compound that reduced *COL1A1* expression at comparable level to sorafenib, a known anti-fibrotic drug, with statistical significance (Fig. 1A). The *COL1A1* suppressive effect was dose-dependent (Fig. 1B), which was also confirmed at the protein level (Fig. 1C). Expression of *PDGFRB*, encoding platelet-derived growth factor receptor-β, was similarly reduced by 10 μM of apigenin (Fig. 1D). Cell viability assessment showed that the compound is not toxic at concentrations below 20 μM (Fig. 1E). Other known liver fibrosis-related genes, *ASMA, TGFB1*, and *TGFBR1*, were not suppressed at the non-toxic concentration (Fig. 1F–H), suggesting that apigenin’s effect is directed to a specific subset of fibrogenesis-related pathways.

### *C1QTNF2* as a potential intracellular target of apigenin

**Figure 2:**
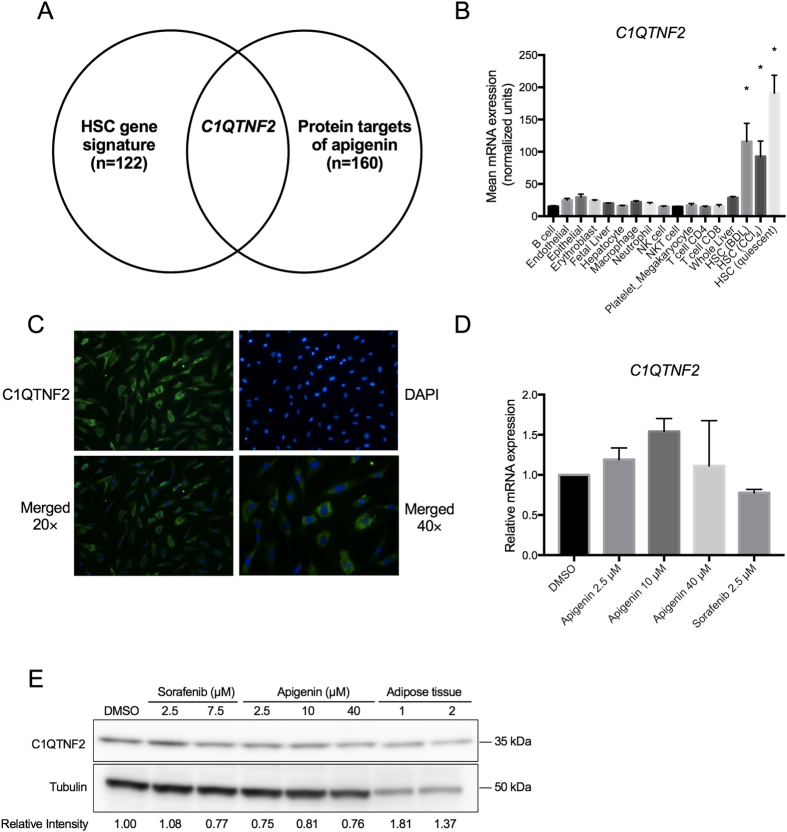
HSC-specific expression of C1QTNF2, a potential target of apigenin. (**A**) Overlap between HSC gene signature^8^ and potential intracellular targets of apigenin^18^. (**B**) *C1QTNF2* expression in a panel of various cell types isolated from mouse livers (expression DNA microarray, n ≥ 3 in each cell type)^8^. BDL: bile duct ligation; CCl4: carbon tetrachloride. *p < 0.001, Tukey’s test. (**C**) Subcellular localization of C1QTNF2 protein in TWNT-4 cells (immunofluorescence staining). (**D**) Modulation of *C1QTNF2* expression by apigenin and sorafenib in TWNT-4 cells (qRT-PCR, n = 2). (**E**) Modulation of C1QTNF2 protein by apigenin and sorafenib in TWNT-4 cells and adipose tissues (Western blotting, n = 1). Relative intensity to tubulin was calculated.

**Figure 3:**
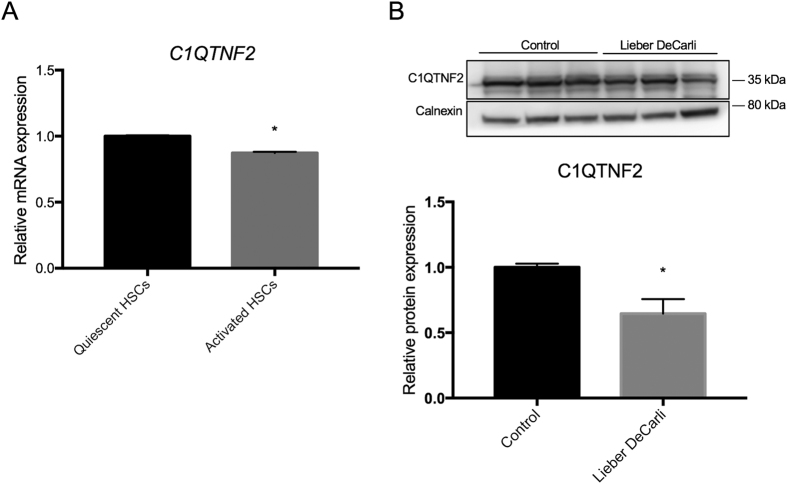
Reduced C1QTNF2 mRNA and protein expression in mouse primary HSCs and ethanol-injured liver. (**A**) *C1QTNF2* expression in freshly isolated (“quiescent”) and 7-day-cultured (“activated”) mouse primary HSCs^13^ (expression DNA microarray, n = 3). *p < 0.001, t-test. (**B**) C1QTNF2 protein expression in livers from mice fed with Lieber DeCarli ethanol-containing diet normalized to calnexin (Western blotting, n = 5). *p < 0.05, t-test. Bar graphs show mean and standard error of mean (SEM) (error bars) of replicated experiments.

**Figure 4:**
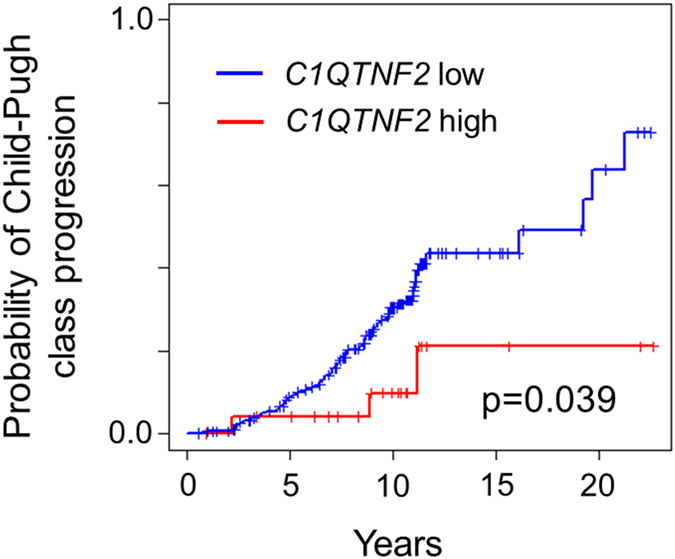
Prognostic association of *C1QTNF2* expression in a clinical HCV cirrhosis cohort. (**A**) clinical cohort of 216 patients with compensated HCV-related cirrhosis^6^ was classified into *C1QTNF2*-high (n = 25) and low (n = 101) groups, and evaluated for association with cirrhosis progression, i.e., progression of Child-Pugh class^21^ from (**A)** to (**B**) or (**C**). P-value was calculated by log-rank test.

Next we sought to identify targets of apigenin in hepatic stellate cells. A 122-gene signature uniquely expressed in HSC^8^ was overlaid on a list of 160 genes encoding intracellular proteins that physically interact with apigenin based on phase display^18^. *C1QTNF2* was identified as the only gene common to the 2 gene lists (Fig. 2A). With 20,354 protein-coding genes in human genome according to NCBI CCDS database (release 21) (www.ncbi.nlm.nih.gov/projects/CCDS), the number of apigenin target genes (160 genes) that could be found within the hepatic stellate cell signature (122 genes) by chance is less than 1 (0.959), although it does not reach statistical significance (p = 0.37, hypergeometric test). To date, C1q and tumor necrosis factor related protein 2 encoded (C1QTNF2) protein is an adipokine that has been identified previously in adipose tissue, where it has effects on lipid metabolism and insulin activity^19^, but upregulation of C1QTNF2 mRNA expression unique to HSC was confirmed in a panel of various cell types presenting in fibrotic liver, which were assembled in our previous study^8^ (Fig. 2B). Furthermore, immunofluorescence staining showed strong cytoplasmic expression of C1QTNF2 in TWNT-4 cells (Fig. 2C). Apigenin treatment did not alter *C1QTNF2* mRNA and protein abundance (Fig. 2D,E). These results suggest that apigenin elicits its COL1A1-suppressive effect in HSC without modulating C1QTNF2 expression levels.. However, apigenin could interfere with the function of C1QTNF2 protein via a physical interaction. Interestingly, baseline *C1QTNF2* mRNA expression was reduced during the process of *in vivo* HSC activation by carbon tetrachloride (CCl_4_) treatment or bile duct ligation (BDL) (Fig. 2B) and *in vitro* culture activation^13^ (Fig. 3A). C1QTNF2 protein expression was similarly reduced in the livers from mouse model of alcoholic injury by Lieber DeCarli ethanol-containing diet for 6 weeks^20^ (Fig. 3B). Furthermore, in a clinical cohort of 216 patients with HCV-related compensated cirrhosis, patients with high *C1QTNF2* expression showed a better clinical outcome of cirrhosis, as measured by Child-Pugh classification^21^ (Fig. 4). These animal model- and clinical cohort-based findings suggest that *C1QTNF2* plays a protective role in cirrhosis progression. If a physical interaction with C1QTNF2 protein is needed for apigenin to elicit its *COL1A1*-suppresive effect as we computationally predict, it may be possible that the status of C1QTNF2 expression can serve as a predictive marker of apigenin responses, which could be clarified in future studies.

## Discussion

Molecular signature-based unbiased and hypothesis-free computational drug discovery has been successfully utilized primarily in cancer and inflammatory diseases^3^. Our study has demonstrated that this strategy can be similarly applied to anti-fibrotic drug discovery. Generation of the query gene signature in transcriptomic profiles of activated HSCs enabled the discovery of candidate compounds specific to the biological context, circumventing the need for costly large compound library screen. The identification of apigenin, already known to be anti-fibrotic in other tissue types such as pancreas^22^,^23^, clearly indicates that our approach is a viable option to discover biologically relevant anti-fibrotic agents in a cost-effective manner.

Apigenin is a flavonoid, abundant in parsley and celery, that has gained interest as a health-promoting agent because of its low intrinsic toxicity^24^. Despite the promising *in vitro* anti-fibrogenic activity comparable to sorafenib^14^, its poor solubility limits optimal *in vivo* biodistribution and needs further biochemical modifications to improve solubility. In addition, apigenin is known to modulate the immune response^18^, although our *in vitro* experimental system did not uncover an immune-related activity, which could be assessed in future studies. C1QTNF2, which we have associated with liver disease severity and prognosis, may be a factor that potentially influences the outcome of apigenin-based therapy in particular, and the biology of hepatic fibrosis in general. C1QTNF2 is a member of C1q/TNF-related proteins that represent an adipokine family less characterized than other well-studied adipocytokines implicated in liver fibrosis, such as adiponectin^19^,^25^. The only known source of C1QTNF2 production is stromal vascular cells in adipose tissue, and the highly restricted sites of production suggests its function is distinct from adiponectin^19^. Indeed, C1QTNF2 does not substitute for adiponectin in caloric restriction^26^. C1QTNF2 is known to be involved in several metabolic processes such as AMP kinase phosphorylation to stimulate glucose uptake in muscle cells^27^ and improvement of insulin and lipid tolerance in diet-induced obese mice^28^. C1QTNF2 may form heteromers with C1QTNF7 and adiponectin^19^. Our study suggets it has a novel role in HSC biology that could maintain the cell’s quiescent state. These findings reinforce the value of computational approaches to drug discovery for hepatic fibrosis, and identify C1QTNF2 as a potential mediator of apigenin’s anti-fibrotic activity.

## Additional Information

**How to cite this article**: Hicks, D. F. *et al*. Transcriptome-based repurposing of apigenin as a potential anti-fibrotic agent targeting hepatic stellate cells. *Sci. Rep.* **7**, 42563; doi: 10.1038/srep42563 (2017).

**Publisher's note:** Springer Nature remains neutral with regard to jurisdictional claims in published maps and institutional affiliations.

## Supplementary Material

Supplementary Information

## Figures and Tables

**Table 1:** Compounds predicted to be antifibrotic by an *in silico* screen with key pharmacological and clinical characteristics.

Drug	Enrichment score	p value	Regulatory approval	Test in human	Known adverse effects	Published anti-fibrotic effect
leflunomide	−0.20	0.002	Yes	Yes^29^	Diarrhea, nausea, alopecia, rash^29^	No
AH-23848	−0.19	0.005	No	Yes^30^	Heartburn, nausea, vomiting^31^	No
xamoterol	−0.18	0.008	No	Yes^32^	Nausea^32^	No
6-benzylaminopurine	−0.18	0.01	No	No	Uncertain	No
irinotecan	−0.17	0.015	Yes	Yes^33^	Bone marrow suppression, diarrhea, alopecia, fatigue, nausea, vomiting^33^	No
hydroxyachillin	−0.17	0.015	No	No	Uncertain	No
aceclofenac	−0.17	0.024	Yes	Yes^34^	Moderate epigastric discomfort and dyspepsia^34^	Yes^35^
methylergometrine	−0.16	0.028	Yes	Yes^36^	Rare Acute Coronary Syndrome, Myocardial Infarction^37^	No
CP-863187^38^	−0.16	0.036	No	No	Uncertain	No
estrone	−0.16	0.028	Yes	Yes^39^	CHD, stroke, breast cancer, PE^39^	No
apigenin	−0.16	0.041	No	Yes^40^	Uncertain	Yes^16^,^17^
PHA-00767505E^41^	−0.16	0.053	No	No	Uncertain	No
digoxigenin	−0.16	0.045	No	No	Uncertain	No
5252917^42^	−0.16	0.031	No	No	Uncertain	No
3-aminobenzamide	−0.16	0.04	No	No	Uncertain	Yes^43^
zuclopenthixol	−0.16	0.042	Yes	Yes^44^	Extra-pyramidal symptoms^44^	No
bucladesine	−0.16	0.054	No	Yes^45^,^46^	Vasodilation^45^,^46^	Yes^47^
erastin	−0.15	0.048	No	No	Uncertain	No

^*^Approval by any international agency.
