# Supplementary material for: Transcriptome-based repurposing of apigenin as a potential anti-fibrotic agent targeting hepatic stellate cells
Source: Sci Rep. 2017 Mar 3;7:42563. doi: 10.1038/srep42563 (PMC5335661; doi:10.1038/srep42563)

Supporting information

Transcriptome-based repurposing of apigenin as a potential anti-fibrotic agent targeting hepatic stellate cells

Daniel F. Hicks, Nicolas Goossens, Ana Blas-García, Takuma Tsuchida, Benjamin Wooden, Michael C. Wallace, Natalia Nieto, Abigale Lade, Benjamin Redhead, Joel T. Dudley, Bryan C. Fuchs, Youngmin A. Lee, Yujin Hoshida, and Scott L. Friedman

Supplementary table 1
HSC activation gene signature in low-dose DEN rats.

Gene symbol (human)	Gene symbol (rat)	t-statistic		Gene symbol (human)	Gene symbol (rat)	t-statistic
Upregulated in cirrhosis				Upregulated in control		
TUBB6	*Tubb6*	27.48		*ECH1*	*Ech1*	-20.44
STMN1	*Stmn1*	16.51		*NUPR1*	*Nupr1*	-15.44
MYADM	*Myadm*	13.24		*RGN*	*Rgn*	-9.53
SERPINA7	*Serpina7*	12.95		*SULT1A1*	*Sult1a1*	-8.93
LAMA5	*Lama5*	12.73		*IDI1*	*Idi1*	-8.93
NEURL3	*Lincr*	12.38		*VCAM1*	*Vcam1*	-7.85
SCAMP2	*Scamp2*	12.16		*NRBF2*	*Nrbf2*	-7.49
KIF22	*Kif22*	9.49		*FAHD1*	*Fahd1*	-6.42
SPC25	*Spc25*	9.27		*FMO5*	*Fmo5*	-6.34
LTBP2	*Ltbp2*	8.87		*ARG1*	*Arg1*	-6.13
SRPX	*Srpx*	8.66		*ZFP36*	*Zfp36*	-5.92
SLC1A5	*Slc1a5*	8.31		*CDO1*	*Cdo1*	-5.89
CDC2	*Cdc2*	8.31		*GNMT*	*Gnmt*	-5.86
FABP4	*Fabp4*	8.29		*CCS*	*Ccs*	-5.77
ZDHHC2	*Zdhhc2*	7.85		*SPRYD4*	*Spryd4*	-5.69
CRYAB	*Cryab*	7.66		*P2RY2*	*P2ry2*	-5.66
PDGFA	*Pdgfa*	7.45		*GLUD1*	*Glud1*	-5.53
SULF2	*Sulf2*	7.36		*HSD17B2*	*Hsd17b2*	-5.21
SLC20A1	*Slc20a1*	7.09		*NDUFV2*	*Ndufv2*	-5.18
SCN3B	*Scn3b*	7.00		*HMGCS1*	*Hmgcs1*	-5.16
FAM3C	*Fam3c*	6.96		*ATF5*	*Atf5*	-4.96
LGALS3BP	*Lgals3bp*	6.90		*WIPI2*	*Wipi2*	-4.93
ATP6V1D	*Atp6v1d*	6.72		*AMD1*	*Amd1*	-4.51
EDNRB	*Ednrb*	6.66		*SLC25A25*	*Slc25a25*	-4.48
PGD	*Pgd*	6.50		*CYB5A*	*Cyb5a*	-4.47
NID2	*Nid2*	6.44		*OPTN*	*Optn*	-4.47
DDIT4L	*Ddit4l*	6.38		*CHDH*	*Chdh*	-4.47
PLAT	*Plat*	6.22		*GPX1*	*Gpx1*	-4.46
C15orf23	*Traf4af1*	6.18		*ZBTB43*	*Zbtb43*	-4.41
PCYOX1	*Pcyox1*	6.11		*HMGCS2*	*Hmgcs2*	-4.37
TUSC3	*Tusc3*	5.99		*SEPHS2*	*Sephs2*	-4.34
MANBA	*Manba*	5.89		*PER2*	*Per2*	-4.32
DAG1	*Dag1*	5.85		*AS3MT*	*As3mt*	-4.29
PLP2	*Plp2*	5.77		*SLC27A5*	*Slc27a5*	-4.28
LBP	*Lbp*	5.75		*LYPLA1*	*Lypla1*	-4.27
DPEP1	*Dpep1*	5.49		*COMMD5*	*Commd5*	-4.25
DCN	*Dcn*	5.38		*DUSP1*	*Dusp1*	-4.25
IFI30	*Ifi30*	5.36		*FMO1*	*Fmo1*	-4.18
PCOLCE	*Pcolce*	5.33		*TAT*	*Tat*	-4.16
NGFRAP1	*Ngfrap1*	5.33		*MACROD1*	*Macrod1*	-4.14
LGALS1	*Lgals1*	5.32		*ACN9*	*Acn9*	-4.07
PLSCR1	*Plscr1*	5.15		*LIN7A*	*Lin7a*	-4.06
EPHX1	*Ephx1*	5.14		*NDRG2*	*Ndrg2*	-4.04
TYMS	*Tyms*	5.13		*C4orf34*	*RGD1311122*	-4.00
KRT19	*Krt19*	5.12		*PAH*	*Pah*	-4.00
CCNG1	*Ccng1*	5.07		*CD14*	*Cd14*	-3.97
PNOC	*Pnoc*	5.05		*CSAD*	*Csad*	-3.93
FBN1	*Fbn1*	4.99		*NR1D1*	*Nr1d1*	-3.92
VEGFC	*Vegfc*	4.84		*FEZ1*	*Fez1*	-3.92
IGFBP7	*Igfbp7*	4.83		*GRPEL1*	*Grpel1*	-3.90
SERPINB1	*Serpinb1a*	4.74		*RND3*	*Rnd3*	-3.87
GJB2	*Gjb2*	4.69		*SLC39A8*	*Slc39a8*	-3.85
HMOX1	*Hmox1*	4.67		*CDK7*	*Cdk7*	-3.80
ITGB1	*Itgb1*	4.66		*PHYH*	*Phyh*	-3.78
SYNJ2	*Synj2*	4.65		*DBI*	*Dbi*	-3.75
PQLC3	*Pqlc3*	4.65		*ADK*	*Adk*	-3.70
LPL	*Lpl*	4.58		*HRSP12*	*Hrsp12*	-3.69
ABCC3	*Abcc3*	4.56		*GLYAT*	*Glyat*	-3.69
PLOD2	*Plod2*	4.55		*MRPL41*	*Mrpl41*	-3.68
PRNP	*Prnp*	4.53		*ACLY*	*Acly*	-3.67
CPZ	*Cpz*	4.51		*FABP1*	*Fabp1*	-3.63
COL4A1	*Col4a1*	4.50		*ACADM*	*Acadm*	-3.62
ATP1B1	*Atp1b1*	4.48		*DNAJA1*	*Dnaja1*	-3.62
ACP6	*Acp6*	4.46		*BRP44L*	*Brp44l*	-3.61
IGFBP2	*Igfbp2*	4.44		*NR1H4*	*Nr1h4*	-3.58
CCNB2	*Ccnb2*	4.41		*GFAP*	*Gfap*	-3.57

COL5A2	*Col5a2*	4.39		*UOX*	*Uox*	-3.56
JAM3	*Jam3*	4.38		*C11orf54*	*RGD1309534*	-3.56
SLC16A3	*Slc16a3*	4.38		*CEBPG*	*Cebpg*	-3.53
FKBP10	*Fkbp10*	4.29		*CXCL11*	*Cxcl11*	-3.51
CYP26A1	*Cyp26a1*	4.28		*THRSP*	*Thrsp*	-3.47
A2M	*A2m*	4.25		*CXCL12*	*Cxcl12*	-3.46
PCP4	*Pcp4*	4.25		*KLF15*	*Klf15*	-3.46
PVR	*PVR*	4.23		*THRB*	*Thrb*	-3.45
SLC7A5	*Slc7a5*	4.21		*SSX2IP*	*Ssx2ip*	-3.44
RAB13	*Rab13*	4.19		*HPD*	*Hpd*	-3.42
CCL21	*Ccl21b*	4.17		*GPHN*	*Gphn*	-3.42
KIFC1	*Kifc1*	4.15		*CROT*	*Crot*	-3.41
APRT	*Aprt*	4.14		*CBX7*	*Cbx7*	-3.39
ALDH1A1	*Aldh1a1*	4.14		*ZNF354A*	*Zfp354a*	-3.38
CD36	*Cd36*	4.12		*GALM*	*Galm*	-3.36
DCTPP1	*Xtp3tpa*	4.09		*DCI*	*Dci*	-3.36
CA2	*Car2*	4.08		*LARP1B*	*RGD1307509*	-3.35
RRM2	*Rrm2*	3.97		*NOX4*	*Nox4*	-3.35
PLD3	*Pld3*	3.96		*PIK3C3*	*Pik3c3*	-3.34
NQO1	*Nqo1*	3.96		*TIMM8B*	*Timm8b*	-3.34
S100A6	*S100a6*	3.92		*BET1*	*Bet1*	-3.34
GPX2	*Gpx2*	3.92		*HSD17B4*	*Hsd17b4*	-3.32
GSTA3	*Yc2*	3.90		*MTERFD2*	*Mterfd2*	-3.31
SNX20	*Snx20*	3.90		*SPATS2L*	*RGD1309930*	-3.30
BOK	*Bok*	3.86		*RPP21*	*Rpp21*	-3.29
CDC20	*Cdc20*	3.84		*SCLY*	*Scly*	-3.28
ADPRH	*Adprh*	3.81		*DECR1*	*Decr1*	-3.27
IGF2R	*Igf2r*	3.80		*UCHL5*	*Uchl5*	-3.26
PLA2G7	*Pla2g7*	3.80		*ESM1*	*Esm1*	-3.26
MYC	*Myc*	3.77		*CA5A*	*Car5a*	-3.25
CTSB	*Ctsb*	3.76		*THUMPD1*	*Thumpd1*	-3.22
GPX3	*Gpx3*	3.74		*ECM1*	*Ecm1*	-3.22
RND1	*Rnd1*	3.73		*BAAT*	*Baat*	-3.18
CCNA2	*Ccna2*	3.71		*ACADL*	*Acadl*	-3.17
RNASE1	*Rnase1*	3.71		*ALDOB*	*Aldob*	-3.16
RGS10	*Rgs10*	3.70		*UGP2*	*Ugp2*	-3.16
LGALS2	*Lgals2*	3.68		*IL1A*	*Il1a*	-3.13
MGP	*Mgp*	3.67		*UPB1*	*Upb1*	-3.13
LTBP4	*Ltbp4*	3.67		*FCGR2B*	*Fcgr2b*	-3.13
FBLN5	*Fbln5*	3.65		*BHMT*	*Bhmt*	-3.11
NUF2	*Nuf2*	3.65		*TIMM8A*	*Timm8a1*	-3.09
MCM6	*Mcm6*	3.60		*IVD*	*Ivd*	-3.08
TSPO	*Tspo*	3.59		*SLC13A3*	*Slc13a3*	-3.08
F2R	*F2r*	3.58		*CAT*	*Cat*	-3.07
DYNLT1	*Dynlt1*	3.58		*ACY3*	*Acy3*	-3.07
RPL31	*Rpl31*	3.57		*MCTS1*	*Mcts1*	-3.06
REEP6	*Reep6*	3.57		*FGL1*	*Fgl1*	-3.02
C8orf85	*Aard*	3.56		*MAOB*	*Maob*	-3.00
RHOB	*Rhob*	3.56		*HSPB1*	*Hspb1*	-2.97
PROCR	*Procr*	3.56		*TEK*	*Tek*	-2.95
LAPTM4B	*Laptm4b*	3.54		*USP2*	*Usp2*	-2.93
TNFSF13	*Tnfsf13*	3.54		*CXCL10*	*Cxcl10*	-2.91
PIR	*Pir*	3.51		*PPP1R3B*	*Ppp1r3b*	-2.91
PLOD1	*Plod1*	3.50		*GNE*	*Gne*	-2.86
AURKB	*Aurkb*	3.50		*COMT*	*Comt*	-2.85
DDAH2	*Ddah2*	3.49		*AKR1D1*	*Akr1d1*	-2.85
RTN1	*Rtn1*	3.48		*HES6*	*Hes6*	-2.84
P2RY13	*P2ry13*	3.48		*CHN1*	*Chn1*	-2.82
CST3	*Cst3*	3.47		*GIMAP4*	*Gimap4*	-2.82
GPC3	*Gpc3*	3.46		*SKP1*	*Skp1*	-2.80
CRYL1	*Cryl1*	3.44		*PCK1*	*Pck1*	-2.79
TAGLN	*Tagln*	3.43		*MLYCD*	*Mlycd*	-2.78
SPON1	*Spon1*	3.42		*STRBP*	*Strbp*	-2.76
HCK	*Hck*	3.41		*APOF*	*Apof*	-2.76
MFAP4	*Mfap4*	3.40		*C10orf10*	*LOC500300*	-2.75
KIAA0101	*Ns5atp9*	3.38		*ALDH3A2*	*Aldh3a2*	-2.74
RBMS2	*Rbms2*	3.34		*ACADVL*	*Acadvl*	-2.72
TUBA1A	*Tuba1a*	3.32		*SEZ6*	*Sez6*	-2.72
KCNN4	*Kcnn4*	3.32		*TINAGL1*	*Tinagl1*	-2.72
OGFRL1	*Ogfrl1*	3.31		*LRRN1*	*Lrrn1*	-2.71
TLR2	*Tlr2*	3.28		*FBP1*	*Fbp1*	-2.70
LCN2	*Lcn2*	3.25		*NDUFS5*	*Ndufs5*	-2.68

ST3GAL2	*St3gal2*	3.23		*ZNF274*	*Zfp110*	-2.68
TSPAN8	*Tspan8*	3.23		*OAT*	*Oat*	-2.68
ABCG2	*Abcg2*	3.22		*TTPA*	*Ttpa*	-2.68
CLIC1	*Clic1*	3.19		*CDC37L1*	*Cdc37l1*	-2.67
PIGS	*Pigs*	3.18		*DPYS*	*Dpys*	-2.67
DDOST	*Ddost*	3.18		*ASL*	*Asl*	-2.66
CNP	*Cnp*	3.16		*LRRN3*	*Lrrn3*	-2.66
MMP12	*Mmp12*	3.15		*PYGL*	*Pygl*	-2.65
TXNRD1	*Txnrd1*	3.13		*CHP*	*Chp*	-2.65
PTGR1	*Ptgr1*	3.12		*FAH*	*Fah*	-2.64
ENPP5	*Enpp5*	3.12		*TSC22D3*	*Tsc22d3*	-2.64
ZFAND2A	*Zfand2a*	3.11		*PFKFB1*	*Pfkfb1*	-2.64
VAV1	*Vav1*	3.09		*NR0B2*	*Nr0b2*	-2.60
CXCR7	*Cxcr7*	3.08		*AKR1C2*	*Akr1c21*	-2.60
TNFRSF12A	*Tnfrsf12a*	3.07		*GUCY1B2*	*Gucy1b2*	-2.60
SPINT1	*Spint1*	3.03		*CBS*	*Cbs*	-2.59
ACSL3	*Acsl3*	3.02		*DMGDH*	*Dmgdh*	-2.59
PMP22	*Pmp22*	3.02		*MYO1B*	*Myo1b*	-2.59
HEPH	*Heph*	3.02		*LOC730107*	*Gcsh*	-2.58
ASPN	*Aspn*	3.01		*TARS*	*Tars*	-2.58
LOXL1	*Loxl1*	3.00		*PCYT2*	*Pcyt2*	-2.58
SLC16A1	*Slc16a1*	3.00		*NFIA*	*Nfia*	-2.58
MAN2B1	*Man2b1*	2.97		*DCAF11*	*Wdr23*	-2.55
MMP14	*Mmp14*	2.96		*DDT*	*Ddt*	-2.54
UNC93B1	*Unc93b1*	2.95		*UFD1L*	*Ufd1l*	-2.53
COL1A1	*Col1a1*	2.94		*ALDH9A1*	*Aldh9a1*	-2.53
AGA	*Aga*	2.94		*SDHA*	*Sdha*	-2.53
FABP2	*Fabp2*	2.94		*AGXT*	*Agxt*	-2.52
DEFB1	*Defb1*	2.93		*MAT1A*	*Mat1a*	-2.50
APOBEC1	*Apobec1*	2.92		*C1orf63*	*RGD1359529*	-2.50
UGT1A6	*Ugt1a6*	2.92		*LTC4S*	*Ltc4s*	-2.49
CMA1	*Cma1*	2.92		*SCP2*	*Scp2*	-2.48
SLC17A3	*Slc17a3*	2.89		*SEC16B*	*Sec16b*	-2.47
MYH10	*Myh10*	2.89		*AKR1E2*	*Akr1cl2*	-2.46
GGCX	*Ggcx*	2.88		*ADHFE1*	*Adhfe1*	-2.46
AOX1	*Aox1*	2.88		*MRPL47*	*Mrpl47*	-2.45
ALDH3A1	*Aldh3a1*	2.87		*C13orf1*	*RGD1306437*	-2.45
SCD	*Scd*	2.87		*SORD*	*Sord*	-2.44
GCLC	*Gclc*	2.86		*HERPUD1*	*Herpud1*	-2.44
tcag7.1260	*Akr1b8*	2.86		*MGC29506*	*RGD1310251*	-2.44
COL1A2	*Col1a2*	2.84		*ASRGL1*	*Asrgl1*	-2.42
ERMP1	*Ermp1*	2.83		*CCBL1*	*Ccbl1*	-2.40
SPARC	*Sparc*	2.83		*FAM82B*	*Fam82b*	-2.40
PSAP	*Psap*	2.82		*LEPREL1*	*Leprel1*	-2.39
CD151	*Cd151*	2.81		*P2RY14*	*P2ry14*	-2.39
CKB	*Ckb*	2.80		*KYNU*	*Kynu*	-2.39
CX3CL1	*Cx3cl1*	2.79		*SUCLG1*	*Suclg1*	-2.39
TUBB	*Tubb5*	2.78		*LIPE*	*Lipe*	-2.38
EMP3	*Emp3*	2.77		*IDH1*	*Idh1*	-2.38
C4orf18	*MGC72614*	2.76		*ERRFI1*	*Errfi1*	-2.37
EHD4	*Ehd4*	2.74		*TUBA4A*	*Tuba4a*	-2.37
RAB3D	*Rab3d*	2.73		*PKLR*	*Pklr*	-2.37
TMEM55A	*Tmem55a*	2.73		*SYNRG*	*Ap1gbp1*	-2.37
CLEC10A	*Mgl1*	2.73		*CIDEC*	*Cidec*	-2.36
NRGN	*Nrgn*	2.72		*UBC*	*Ubc*	-2.36
EMP1	*Emp1*	2.72		*TMEM204*	*Tmem204*	-2.35
LTBP1	*Ltbp1*	2.71		*HADH*	*Hadh*	-2.35
ERGIC2	*RGD1310606*	2.70		*LONP1*	*Lonp1*	-2.35
TMEM184C	*Tmem184c*	2.69		*ANKRD46*	*Ankrd46*	-2.35
PDLIM7	*Pdlim7*	2.69		*TST*	*Tst*	-2.33
SLC6A6	*Slc6a6*	2.67		*ECHS1*	*Echs1*	-2.33
PLA1A	*Pla1a*	2.66		*GZMA*	*Gzma*	-2.33
FBLIM1	*Fblim1*	2.66		*HAAO*	*Haao*	-2.31
PLEC1	*Plec1*	2.65		*TIMM10*	*Timm10*	-2.31
DDX39	*Ddx39*	2.65		*PDK1*	*Pdk1*	-2.30
PLOD3	*Plod3*	2.65		*LHPP*	*Lhpp*	-2.29
DAP	*Dap*	2.65		*SPIN1*	*Spin1*	-2.28
TMED9	*Tmed9*	2.62		*HSF2*	*Hsf2*	-2.28
FXYD6	*Fxyd6*	2.62		*DNASE1L3*	*Dnase1l3*	-2.27
PABPC1	*Pabpc1*	2.61		*GHITM*	*Ghitm*	-2.27
CPA3	*Cpa3*	2.60		*ACOX2*	*Acox2*	-2.26
ALOX5AP	*Alox5ap*	2.60		*GALT*	*Galt*	-2.26

ZDHHC13	Zdhhc13	2.59		C5orf44	RGD1306583	-2.26
TPM4	Tpm4	2.59		GLS2	Gls2	-2.26
HMGN2	Hmgn2	2.59		HGD	Hgd	-2.25
BCAT2	Bcat2	2.58		APOA2	Apoa2	-2.25
RARRES1	Rarres1	2.58		EHD3	Ehd3	-2.25
ARPC1B	Arpc1b	2.55		HAGH	Hagh	-2.24
ANXA2	Anxa2	2.55		KLF9	Klf9	-2.24
H2AFZ	H2afz	2.55		CALD1	Cald1	-2.24
RASL11B	Rasl11b	2.54		MAOA	Maoa	-2.24
AQP8	Aqp8	2.53		HADHB	Hadhb	-2.23
GSR	Gsr	2.53		DECR2	Decr2	-2.23
CD74	Cd74	2.53		ADI1	Adi1	-2.23
ABP1	Abp1	2.52		UNG	Ung	-2.23
VKORC1L1	Vkorc1l1	2.52		ABHD14B	Abhd14b	-2.23
YWHAH	Ywhah	2.51		ALAD	Alad	-2.22
MS4A2	Ms4a2	2.50		GUCY1B3	Gucy1b3	-2.21
GLG1	Glg1	2.50		TSPAN12	Tspan12	-2.21
TGFB1	Tgfb1	2.49		PPAP2B	Ppap2b	-2.21
ADH1C	Adh1	2.49		TRAP1	Trap1	-2.20
TMEM111	Tmem111	2.48		CTH	Cth	-2.20
TECR	Gpsn2	2.47		PECI	Peci	-2.20
GSN	Gsn	2.47		RAPGEF4	Rapgef4	-2.20
PGRMC1	Pgrmc1	2.47		HSD17B8	Hsd17b8	-2.18
TMEM43	Tmem43	2.47		SULT1C2	Sult1c2	-2.17
FAM105A	Fam105a	2.45		CYP4B1	Cyp4b1	-2.17
CHST1	Chst1	2.44		ADAM23	Adam23	-2.17
DCAKD	Dcakd	2.44		C21orf59	RGD1306954	-2.16
NME1	Nme1	2.43		CPT2	Cpt2	-2.15
GCLM	Gclm	2.43		CYP7A1	Cyp7a1	-2.15
TAPBP	Tapbp	2.41		ACAA2	Acaa2	-2.15
DPP7	Dpp7	2.38		NR1I2	Nr1i2	-2.15
YIPF1	Yipf1	2.38		PTPN21	Ptpn21	-2.15
FCER1A	Fcer1a	2.38		ARFGAP2	Arfgap2	-2.14
ANXA1	Anxa1	2.36		HMGCL	Hmgcl	-2.13
CXADR	Cxadr	2.36		GSTT2	Gstt2	-2.12
IMPDH2	Impdh2	2.35		HACL1	Hacl1	-2.11
TAGLN2	Tagln2	2.34		ETFA	Etfa	-2.09
RHOG	Rhog	2.34		DHRS4	Dhrs4	-2.09
WDR1	Wdr1	2.34		IGFBP1	Igfbp1	-2.08
PPAP2C	Ppap2c	2.33		RAB9A	Rab9a	-2.07
PLXNB2	Plxnb2	2.32		OAF	Oaf	-2.07
CYBB	Cybb	2.30		LAP3	Lap3	-2.06
LCMT1	Lcmt1	2.29		NFIL3	Nfil3	-2.05
MTTP	Mttp	2.29		CREM	Crem	-2.05
AFG3L2	Afg3l2	2.27		TTC36	Ttc36	-2.05
ACSM2A	Acsm2	2.26		C6orf145	RGD1311307	-2.04
KRT8	Krt8	2.26		AKR1C3	Akr1c18	-2.04
FXYD2	Fxyd2	2.25		PHYHD1	Phyhd1	-2.03
FYN	Fyn	2.25		USO1	Uso1	-2.03
SOCS2	Socs2	2.24		ACOX1	Acox1	-2.02
RTKN	Rtkn	2.24		PER1	Per1	-2.01
IFNGR1	Ifngr1	2.22		IGFBP3	Igfbp3	-2.01
FSTL1	Fstl1	2.22		OTC	Otc	-2.00
FUCA2	Fuca2	2.22		SAA4	Saa4	-2.00
CCND1	Ccnd1	2.20		PEX7	Pex7	-1.98
ARRDC1	Arrdc1	2.19		PTPRR	Ptprr	-1.97
NUP210	Nup210	2.19		PPIF	Ppif	-1.97
SCPEP1	Scpep1	2.18		HMGN1	Hmgn1	-1.94
GSTP1	Gstp2	2.17		RCBTB2	Rcbtb2	-1.94
TALDO1	Taldo1	2.17		GAMT	Gamt	-1.93
HLA-DRB1	RT1-Db1	2.15		HAO2	Hao2	-1.92
THBS4	Thbs4	2.14		SELENBP1	Selenbp1	-1.92
SPP1	Spp1	2.13		C1orf174	RGD1304567	-1.89
H2AFY	H2afy	2.12		DNAJA3	Dnaja3	-1.89
UGT2B17	Ugt2b5	2.07		NIT1	Nit1	-1.88
NUDCD2	Nudcd2	2.07		DAO	Dao	-1.86
PYCRL	Pycrl	2.06		BMF	Bmf	-1.86
CXCR4	Cxcr4	2.06		PLEKHF1	Plekhf1	-1.85
SEMA4A	Sema4a	2.05		RXRG	Rxrg	-1.85
CXCL16	Cxcl16	2.04		UROD	Urod	-1.84
HLA-DQA1	RT1-Ba	2.03		BAG3	Bag3	-1.84
AADAC	Aadac	2.01		OR51E2	Olr59	-1.83

PPP1R14B	*Ppp1r14b*	1.99		*NDUFV1*	*Ndufv1*	-1.83
CLDN5	*Cldn5*	1.99		*CYP1A2*	*Cyp1a2*	-1.82
HN1	*Hn1*	1.96		*ETFDH*	*Etfdh*	-1.82
BASP1	*Basp1*	1.94		*GFRA1*	*Gfra1*	-1.82
APLNR	*Aplnr*	1.93		*HSPB8*	*Hspb8*	-1.82
SLC9A1	*Slc9a1*	1.92		*APOM*	*Apom*	-1.81
PLVAP	*Plvap*	1.90		*OPLAH*	*Oplah*	-1.81
CD24	*Cd24*	1.86		*CYP2F1*	*Cyp2f4*	-1.81
TSPAN31	*Tspan31*	1.78		*NIPSNAP1*	*Nipsnap1*	-1.81
RHBG	*Rhbg*	1.77		*BPHL*	*Bphl*	-1.81
NHEJ1	*Nhej1*	1.75		*ATP5L*	*Atp5l*	-1.81
TMX2	*Tmx2*	1.65		*AADAT*	*Aadat*	-1.80
CES2	*LOC498940*	1.64		*ACOT12*	*Acot12*	-1.79
SLC6A9	*Slc6a9*	1.57		*EGR1*	*Egr1*	-1.78
MKNK2	*Mknk2*	1.50		*SRA1*	*Sra1*	-1.78
TMEM19	*Tmem19*	1.46		*EPHX2*	*Ephx2*	-1.77
NEFL	*Nefl*	1.22		*PSMD9*	*Psmd9*	-1.77
				SLC25A10	*Slc25a10*	-1.75
				PECR	*Pecr*	-1.75
				TFPI2	*Tfpi2*	-1.75
				SECTM1	*Sectm1a*	-1.75
				SLC35C2	*Slc35c2*	-1.75
				C16orf5	*RGD1310686*	-1.74
				RNF141	*rnf141*	-1.74
				ACAT1	*Acat1*	-1.74
				CEBPA	*Cebpa*	-1.74
				DLD	*Dld*	-1.73
				GYS2	*Gys2*	-1.73
				NAPRT1	*Naprt1*	-1.72
				PTH1R	*Pthr1*	-1.72
				ACAT2	*Acat2*	-1.71
				FBXO9	*Fbxo9*	-1.70
				HNF1B	*Hnf1b*	-1.70
				ELMO3	*Elmo3*	-1.70
				NAMPT	*Nampt*	-1.68
				PCBD1	*Pcbd1*	-1.68
				AARS	*Aars*	-1.66
				NEGR1	*Negr1*	-1.66
				COQ10B	*Coq10b*	-1.66
				SORBS2	*Argbp2*	-1.65
				PLEKHB1	*Plekhb1*	-1.65
				QDPR	*Qdpr*	-1.65
				ACAA1	*Acaa1*	-1.64
				CDH17	*Cdh17*	-1.64
				ZC3H15	*Zc3h15*	-1.64
				SERPINB6	*Serpinb6a*	-1.63
				KMO	*Kmo*	-1.63
				PDK2	*Pdk2*	-1.62
				COX7B	*Cox7b*	-1.61
				DPP4	*Dpp4*	-1.61
				TXN2	*Txn2*	-1.60
				SLC25A1	*Slc25a1*	-1.60
				PAWR	*Pawr*	-1.58
				DEPDC7	*Depdc7*	-1.58
				AHCY	*Ahcy*	-1.57
				UBFD1	*Ubfd1*	-1.57
				CHPT1	*Chpt1*	-1.57
				WFDC1	*Wfdc1*	-1.56
				RCAN1	*Rcan1*	-1.55
				ADCK5	*Adck5*	-1.55
				PC	*Pc*	-1.55
				PXMP2	*Pxmp2*	-1.55
				BBOX1	*Bbox1*	-1.55
				GCK	*Gck*	-1.54
				MRPL16	*Mrpl16*	-1.53
				MTHFS	*Mthfs*	-1.52
				STK16	*Stk16*	-1.52
				PTMS	*Ptms*	-1.51
				ABAT	*Abat*	-1.49
				PMPCA	*Pmpca*	-1.49
				APCS	*Apcs*	-1.48
				HYAL2	*Hyal2*	-1.44

				HSD17B10	*Hsd17b10*	-1.44
				APBA3	*Apba3*	-1.43
				MECR	*Mecr*	-1.43
				RBM47	*RGD1359713*	-1.40
				NFIB	*Nfib*	-1.38
				PSMA1	*Psma1*	-1.38
				HINT3	*Hint3*	-1.36
				ADRA1B	*Adra1b*	-1.36
				HADHA	*Hadha*	-1.32
				ABTB2	*Abtb2*	-1.31
				MLLT3	*Mllt3*	-1.31
				SLC22A5	*Slc22a5*	-1.30
				GPD1	*Gpd1*	-1.25
				RAB3IP	*Rab3ip*	-1.25
				PIK3C2G	*Pik3c2g*	-1.23
				ST6GALNAC3	*St6galnac3*	-1.22
				ADIPOR1	*Adipor1*	-1.14
				AKR7A2	*Akr7a2*	-1.10
				DDAH1	*Ddah1*	-1.08
				ARHGAP8	*Arhgap8*	-1.07

HSC: hepatic stellate cell, DEN: diethylnitrosamine.

Figure 1C: Collagen 1

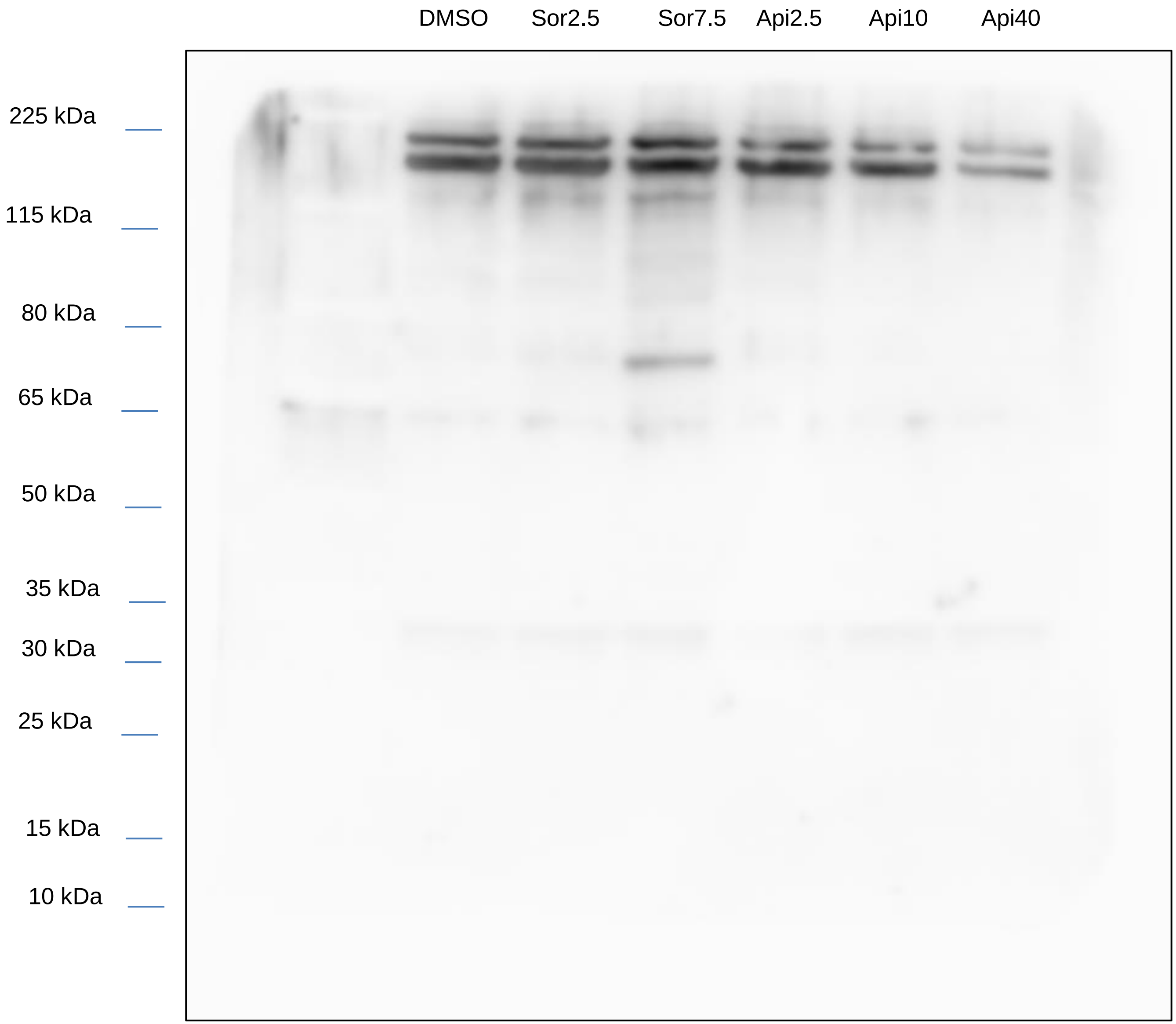

Figure 1C: GAPDH

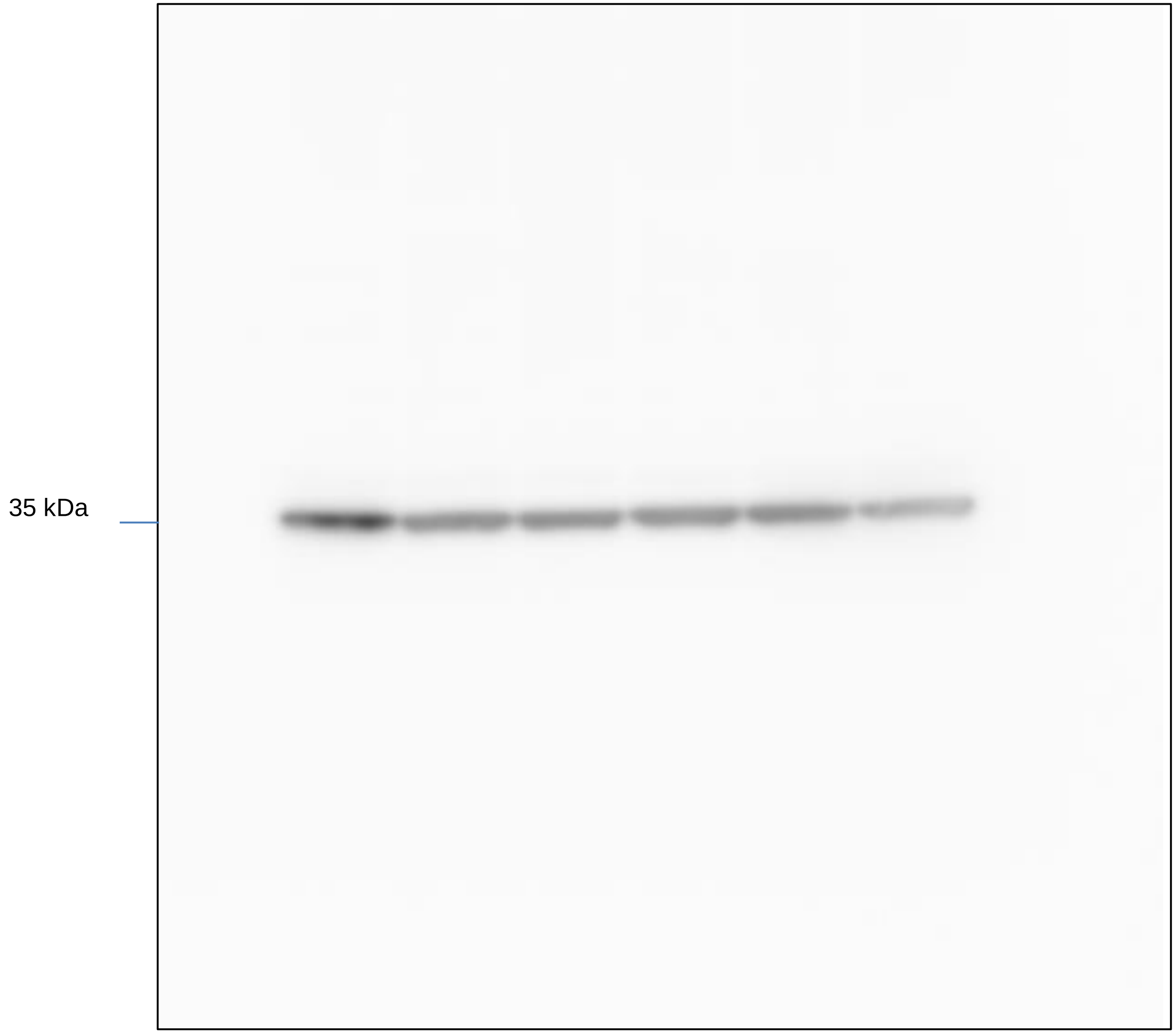

Figure 2E: C1QTNF2

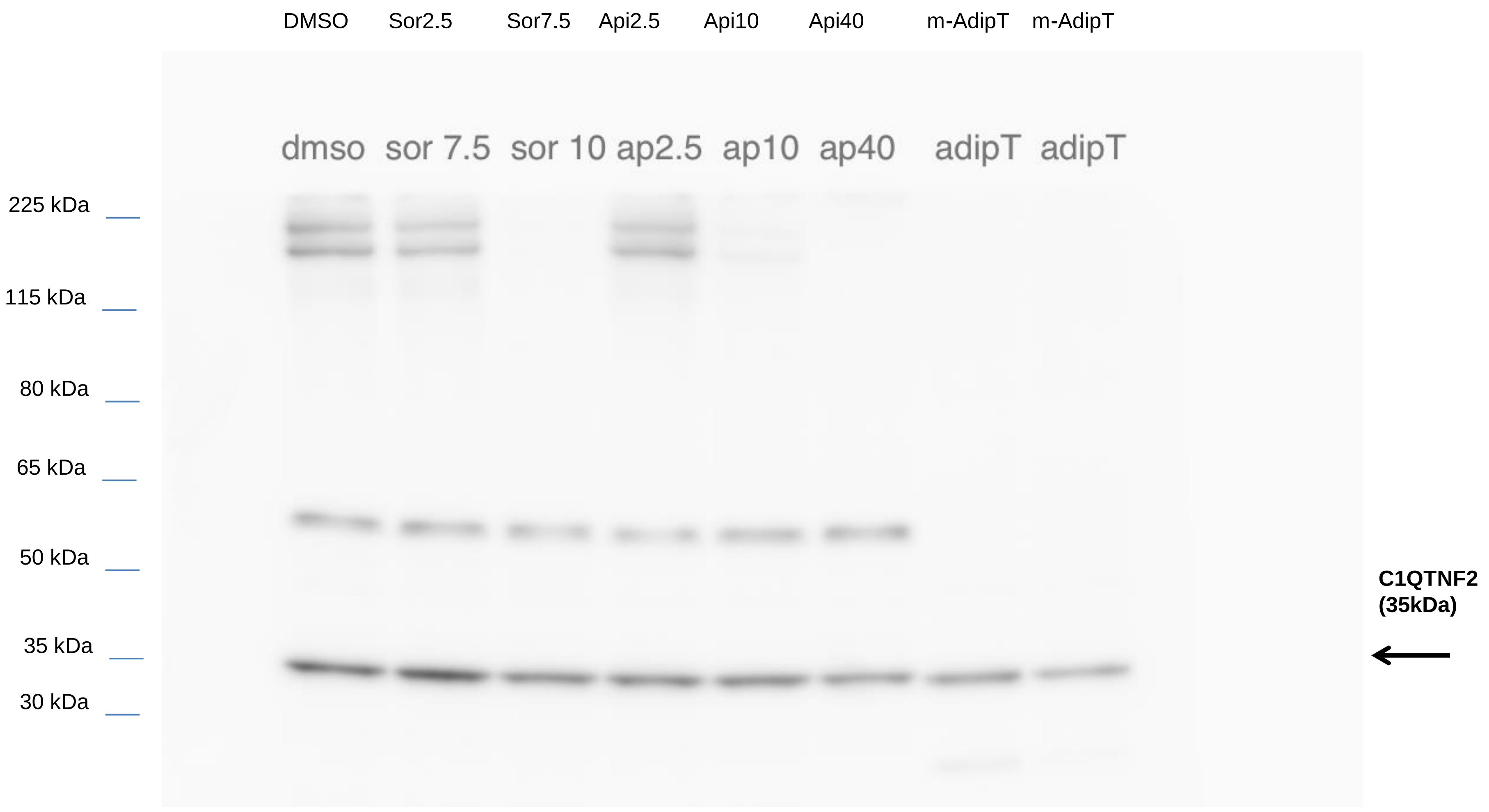

Sorafenib concentrations were mislabeled in the original image

Figure 2E: Tubulin

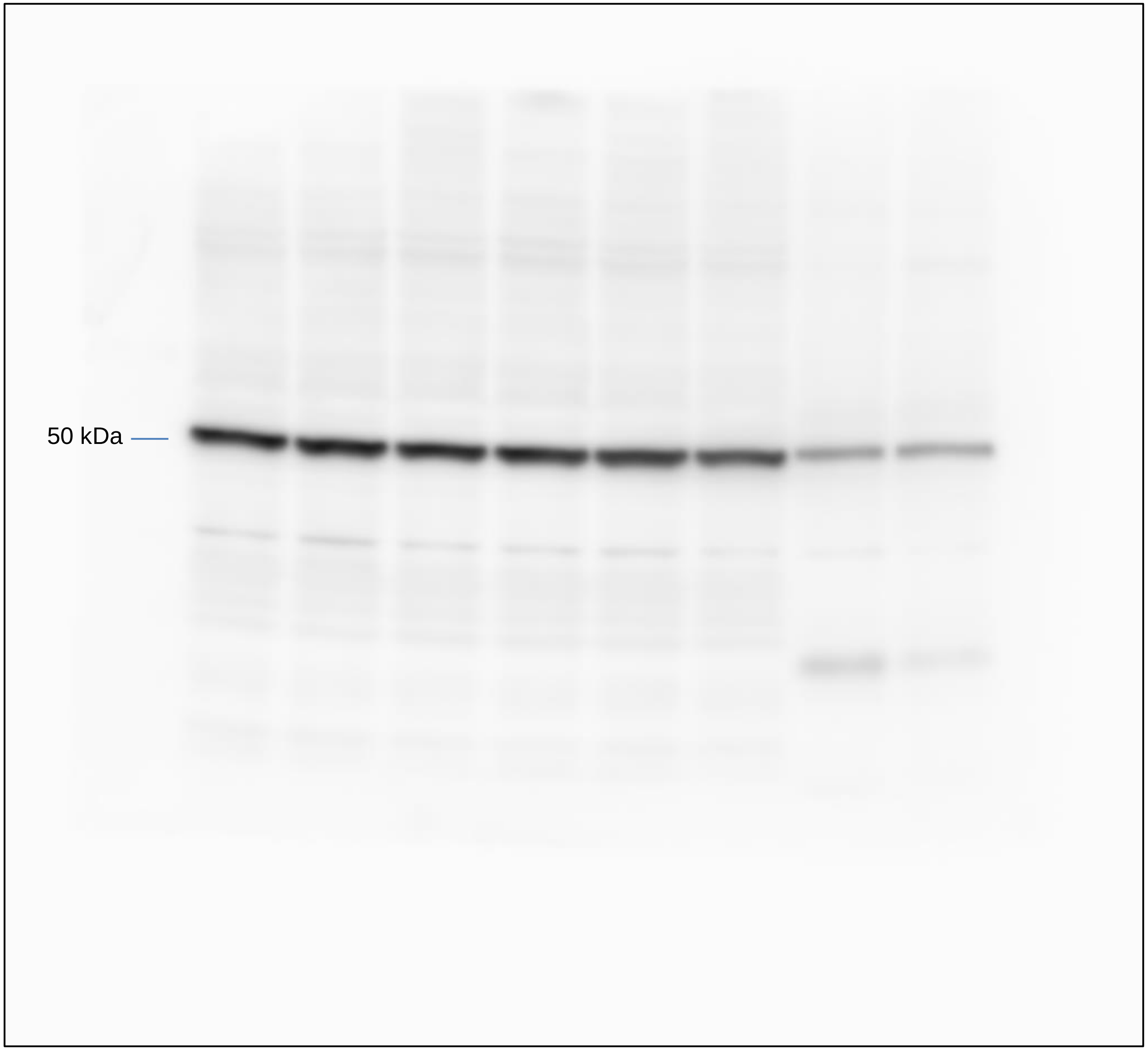

Figure 3B: C1QTNF2

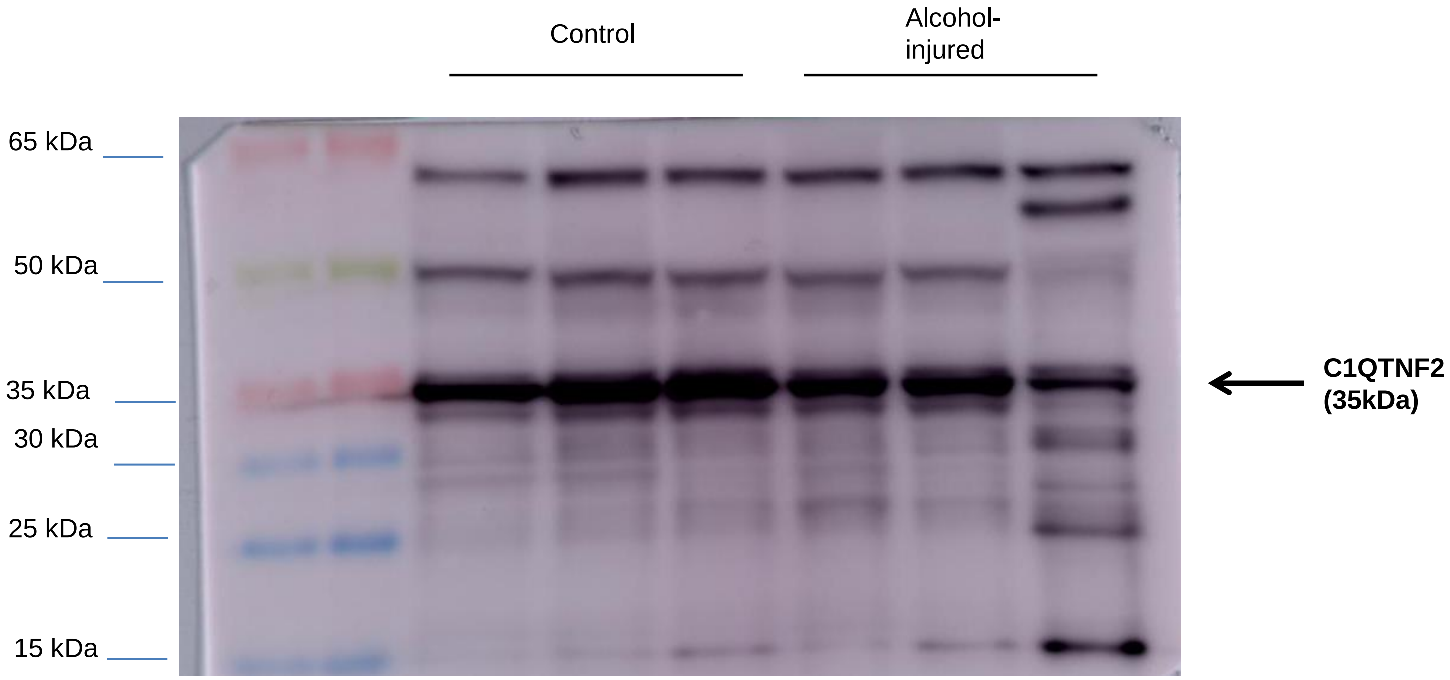

Figure 3B: Calnexin

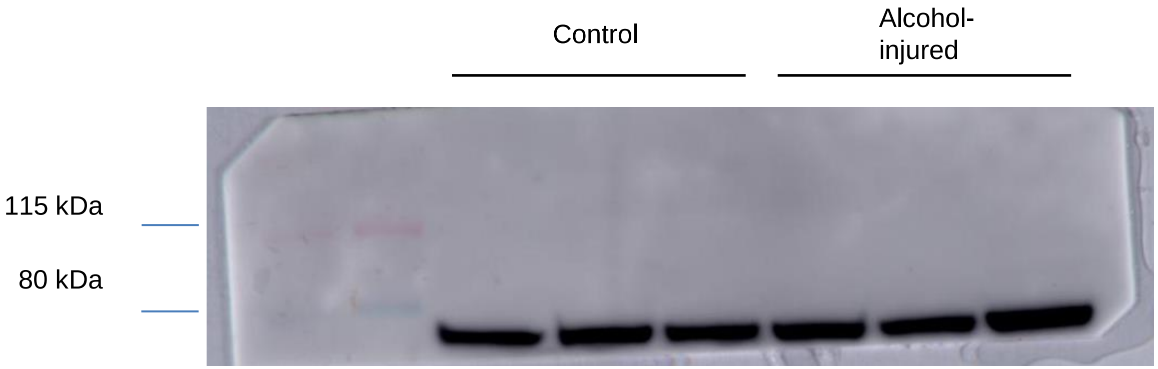

Supplement: Supplementary Information [file srep42563-s1.pdf]
